# Indents

**Published:** 1914-08-15

**Authors:** 


### INDENTS.

☞Brief Articles of Technical or Scientific Value will receive notice and comment. Contributions invited.

*Gift for Hospital.* Henry Ford the Automobile Manufacturer has offered to build a three million dollar hospital for Detroit. We trust some thought will be given to a department for Oral Surgery.

*Filing Small Wire Rods in a Lathe.* Reducing the diameter of a small wire rod by filing, while it is turning in a lathe, is a difficult thing to do, as the pressure of the file on one side bends the rod. The filing may be easily accomplished by using two files. In this manner almost any amount of pressure can be applied by squeezing the files together, without danger of bending the rod.—*Popular Mechanics.*

*Economic Sterilizer for Dental Instruments of Small or Medium Size.* In a dry, sterilized, large glass bottle with wide mouth and glass stopper, a layer of trioxymethylene tablets is placed vertically covered with an even layer of raw sterilized cotton, such as is used in bacteriological work, and this cotton stratum covered with a layer of coarse-grained investment compound about 2 mm. thick. The formaldehyd vapor given off by the tablets will satisfactorily sterilize for several months such instruments as are too delicate to tolerate such means of sterilization as boiling, the autoclave, etc. Sounds, hypodermic needles, surgical or root-canal reamers, broaches, ivory or bone spatulas, and engine handpieces can thus be sterilized effectively at a very low cost.—Raynal, *Sud-est Dentaire.*

*Dental Infection.* I believe that the toxins and living bacteria which pass to the stomach are not infrequently responsible for the failure of the secretion of hydrochloric acid, because on cessation of the active supply of large amounts of bacteria, hydrochloric acid has again been observed in the stomach, and the long present failure on the part of the stomach to digest has gradually given place to a fairly normal digestive process unaided by recourse to the exhibition of hydrochloric acid or other digestents. I further believe that the constant absorption of organisms growing in lesions about the teeth and on the tooth's surfaces themselves are responsible to a greater extent for malnutrition and chronic dyspepsia than any other single cause.

The mouth is the constant habitat of many destructive organisms and offers the best culture media possible for their growth. The passage of an organism from one living being to another keeps these organisms in a constant state of change, exalting or depressing their pathogenic possibilities, dependent upon whether the living being to which they are transferred is relatively highly immune or has no immunity, and the animal passage is being made by the pathogenic bacteria. They gain entrance into the mouth by the following means: the inhalation of the dust of the streets and living rooms, foods, fruits, drinks (milk and water), finger tips, kissing, use of common drinking cups, etc. The constantly changing reactions of the mouth from alkaline to acid favors transmutation as well as animal passage, in that it inspires exalted activity on the part of the organism. Oxygen tension is always reduced just in proportion as the organisms find their way into root canals and pyorrhea pockets, thus making possible changes in organisms which induce them to attack one tissue or another. Clinincal observation is responsible for the belief that almost every individual who reaches manhood or womanhood has one or more blind alveolo-dental abscesses to his or her credit, and that these abscesses and pockets contain streptococci among other organisms. Ninety per cent. of the adult population presents le ions which range all the way from a mild gingivitis to deep blind pyorrhea pockets around their teeth. Tooth root surface in pyorrhea pockets is always more or less coated with living micro-organisms constantly ready to make incursions either into pulp chambers or into the tissue surrounding the roots, where entry into the circulation is rapid, easy and constant; it is this constancy of the supply which eventually breaks down immunity.—T. B. Hartzell, in *Journal of Allied Societies*, June 1914.

*How to Fill Root Canals.* A sense of pressure noted by the patient will determine whether the filling has reached the apical foramen (which must be by a slight sensation of pressure), but we must also be able to determine whether this pressure is from the filling or air. First, apply the rubber dam and dessicate the root canals. Second, flood the pulp canal with chloroform and by the movement of the broach back and forth in the canal air bubbles will escape. Afterwards work chlora-percha in the canal. A new danger arises here, i. e., in the act of removing the broach, air will rush into the pulp canal. This can be avoided by seizing the broach with pliers and drawing the broach slowly out, leaving gutta-percha in its place. Then follow with the gutta-percha point, forcing the latter gently till a slight pressure is noted.—Dr. Woolley, in *Review*.

*The Constitution of Metals.* The mysteries of cohesive gold fillings, the behavior of certain metallic alloys or mixtures, and the anomalous results of pressure casting have led Dr. W. Rosenhain and Mr. D. Ewen to advance a theory on intercrystalline cohesion, which is as follows:

The theory is that the crystals of which metals are built up are held or "cemented" together by an extremely thin layer of amorphous or non-crystalline material chemically identical with the substance of the metal or alloy in question, but in a widely different physical state. The amorphous condition of this intercrystalline layer is regarded as being identical with or at least closely analogous to the condition of a very greatly undercooled liquid, which has remained in that condition in the minute interstices which occur where adjacent crystals meet one another in various orientations. The curve connecting temperature and strength ot such a material would naturally be continuous, while that of crystallizing material would show a discontinuity at the crystallizing point, and the two curves would intersect at a point corresponding to some temperature below the crystallizing temperature. This indicates that at the temperature corresponding to the point of intersection the cement and the crystals have the same strength, but that at other temperatures one or other would be more considerably affected under strain. If the intersection temperature and the solidifying temperature of a metal or alloy are wide apart, then at temperatures just below the solidifying point one should find the cement considerably weaker than the crystals, and under fracture at least for slow straining, the fracture should not be of the intercrystalline type, but should occur without any material deformation of the crystals themselves, even if the crystals are those of extremely ductile metals.

It is pointed out that the general fact that all metals become extremely weak and brittle at temperatures near their melting-points is thus explainable by the amorphous cement theory.

The authors discuss the question as to whether the intercrystalline materia' may not consist of films of eutectic alloys formed with traces of impurities in the metals, but consider the evidence to be much more favorable to the theory they advance.—*British Dental Journal.*

*Alundum Polishing Powder.* Silica or chalk used for polishing teeth (after cleaning them with pumice) leaves a comparatively dull surface compared to that which levigated alundum imparts.

The use of this material for polishing purposes was discovered and given to the dental profession by the writer. Alundum is a fused oxide of alumina, which occurs in nature as the mineral corundum, an abrasive that our grinding wheels were formerly made of. The sapphire, the Oriental topaz, amethyst and emerald are composed of this material in well-crystallized forms. Colored by various metallic oxides alundum is an abrasive made by an electro chemical process; as prepared for polishing teeth it is an amorphous powder. All powders that polish should be amorphous, that is formless or of irregular shape. If an abrasive is of regular or crystalline structure is possesses cutting or grinding qualities, as emery for instance. Such material is excellent for cutting down and smoothing metals, but should not be used for polishing teeth. This also is true of carborundum, adamite, crystolon, silica and cuttle fish (which latter material is always finely ground silica, notwithstanding its label).

This amorphous powder is obtained by crushing and grinding large fused blocks of alundum under water, and is the finely ground, collodial alundum, that is found floating on the top of the grinding trough. The material is collected, washed, and all traces of iron (which may have entered from the jaws of the crusher), removed with an electro magnet.

A small amount of the alundum is placed in a dish and moistened, this paste is then rubbed on the teeth, coating two or three at one time. A napkin should be used to keep the teeth dry, while a very soft polishing brush should be used, run at high speed, taking care not to hold the brush in contact with the tooth long enough to heat it unduly. This material is prepared by the Norton Company, of Worcester, Mass., and should be free from iron or any metallic oxides. It should float, to be suitable for dental use. In larger form alundum is the hardest grinding stone known, and will easily cut carborundum. At the present time the writer is trying small dental engine stones for preparing cavities made of this material, and believes with a feldspar bonding material this stone should cut as well wet as it does dry.—Frederick H. Nies, D. D. S., in *Journal of Allied Societies.*

*Mixing Synthetic Cement.* After a mix of synthetic has been obtained of proper stiffness and consistency, gather it quickly in a ball on the slab and tap it gently several times with the agate spatula. This will develop a fine homogeneous consistency.—Arthur G. Smith, in *Review*.

*Impression of Irregular Teeth.* This is a method of taking impressions for crown or bridge. Warm a piece of sheet paraffine and with the fingers mould it over the adjoining teeth, being careful to bring it not quite to the margins. You want a clear, perfect impression of these margins. Have patient, or assistant, lightly hold in place while plaster is being mixed. The wax will pull off with impression, preventing sticking in crevices or undercuts. Pour impression with wax in place, separate, then remove wax. You have perfect model of adjoining margins for fitting crown or other work.—Dr. F. L. Wright, in *Summary*.

*Facilitating the Taking of Impressions.* If single teeth remain in the mouth, distortion of the plaster cast in separating the cast from the plaster impression can be avoided by first taking an impression with modeling compound, which, while it is still soft, is widened either with a finger or an instrument handle at the places which will offer difficulties in separating. After this enlarged modeling compound impression has hardened, which can be hastened by dipping into cold water, it is filled with plaster, and the final impression is taken. This impression is easy to separate from the cast, as the places where teeth are left are marked by the projecting plaster. These teeth can be further safeguarded against breaking by inserting before pouring the cast, little match-sticks into the depressions left by the remaining teeth. An enlarged modeling compound impression is of great service also in edentulous cases, especially if a high palate is present. In taking an impression of a few teeth in crown and bridge-work, it is very practical to take a preliminary modeling compound impression, which, after having been suitably widened, takes the place of a partial impression tray.—*Zahnarztliche Orthopadie und Prothese.*

*Locating Root Canals.* A very useful method for locating difficult root-canals is as follows: After the cavity has been properly excavated, a pellet of cotton is dipped into a 40 or 50 per cent. solution of sulfuric acid and placed into the pulp chamber for one-half to one minute. The rubber dam in this case should be applied. The pellet is then removed and the acid is neutralized by the application of sodium bicarbonate. The cavity is then syringed with water and dried. Another pellet of cotton is dipped into tincture of iodine and inserted into the cavity. This pellet is also allowed to remain there for from one-half to one minute. Upon removing the pellet, the appearance of black spots will indicate the orifices of the root-canals. The purpose of the sulfuric acid is to dissolve all debris and disintegrated matter, clearing the orifices, while the tincture of iodine strains the orifices black, thus rendering them readily noticeable.—J. A. Klein, *The Acorn.*

*Unslaked Lime for Desensitizing Hypersensitive Dentin.* For the desensitizing of hypersensitive dentin, Calvo recommends the application of the rubber dam, drying the hypersensitive cavity by some simple means, yet leaving enough moisture in the cavity to slake a small amount of unslaked lime. A sensation of heat is perceived which, however, disappears readily. —*Sud-Est Dentaire.*

*The Selection and Application of Flux in Soldering Operations.* Borax can be used in two forms, viz., as a vitrified powder obtained by driving off the water of crystallization from ordinary borax, and calcining the resultant clear mass, and as a mixture with water made by rubbing a piece of borax on a moist slab. Each of these has its good points. The powder being thoroughly dry, does not swell when heat is applied to it, so that even if it should manage to get in between a tooth and its backing no crack will result; here it is distinctly better than its ordinary moist rival. A combination of the two, however, seems to answer best. The surfaces to be soldered, having been thoroughly cleaned, should be sparingly painted with some of the moist borax mixed very thick, care being taken not to touch the investment, and over this some of the powder should be dusted. The piece may then be dried and soldered in the usual way, and if any more flux be required during the operation, the powder should obviously have the preference. —G. J. Goldie, *Ash's Canadian Monthly Circular.*

*Accurate Morsal Surfaces in Porcelain Crowns.* This is a process of reproducing the sulci and the mesial and distal coronal prominences in porcelain crowns. The work is done on articulated amalgam models. Emphasis is laid on the importance of restoring the eminences as a means of properly distributing food and preventing same from crowding into approximal spaces. The details of the method are as follows:

Coping that can be removed from articulated model or coping on jacket crowns preparation articulated is built up with modeling compound just short of occluding space for cusps and marginal ridges. This is left flat on top, oiled and warm modeling compound placed on the same and occlusal articulation made by triturating in anatomical articulator. From this a button of the modeling compound is carved containing all the fine grooves, sulci, indication of buccal and approximal contour and marginal ridges. The button is placed in small amount of plaster for female die from which a male die is again made in modeling compound of more generous diameter than original button, to facilitate handling, etc. From this die a matrix is stamped (plat. 1-1000) reproducing all the fine carvings of original button. A button of porcelain is baked in this cup of platinum and the platinum is then stripped of the buccal and approximal periphery as high as possible without stripping the matrix from the fine carvings in the occlusal surface. The button is then put in its position on the articulator and the crown is baked between the coping as a matrix on the one side and the matrix of the occlusal surface of the porcelain on the other, producing a posterior porcelain jacket or Richmond crown as perfect in detail as one is able to carve in any material, no attention having had to be given to shrinkage of porcelain or any of the inconveniences attending the usual carved porcelain crown.—Dr. A. L. LeGro, in *Summary*.

